# Pneumonitis after normofractionated radioimmunotherapy: a method for dosimetric evaluation

**DOI:** 10.1186/s13014-024-02561-z

**Published:** 2024-11-22

**Authors:** Kim Melanie Kraus, Caroline Bauer, Lisa Steinhelfer, Benedikt Feuerecker, Juliana Cristina Martins, Julius Clemens Fischer, Kai Joachim Borm, Jan Caspar Peeken, Denise Bernhardt, Stephanie Elisabeth Combs

**Affiliations:** 1 Department of Radiation Oncology, School of Medicine and Klinikum rechts der Isar, Technical University of Munich (TUM), 81675 Munich, Germany; 2 Institute of Radiation Medicine (IRM), Helmholtz Zentrum München (HMGU) GmbH German Research Center for Environmental Health, 85764 Neuherberg, Germany; 3 Partner Site Munich, German Consortium for Translational Cancer Research (DKTK), 80336 Munich, Germany; 4 Department of Radiology, School of Medicine and Klinikum Rechts der Isar, Technical University of Munich (TUM), 81675 München, Germany; 5 Department of Radiology, University Hospital Munich, LMU Munich, 81377 Munich, Germany; 6 Bavarian Cancer Research Center (BZKF), Technical University Munich, Munich, Germany

**Keywords:** Immunotherapy, Radioimmunotherapy, Radiation pneumonitis, Lung cancer, Immune checkpoint inhibition, Dose volume histogram

## Abstract

**Background:**

Post-Therapy-Pneumonitis (PTP) is a critical side effect of both, thoracic radio(chemo)therapy (R(C)T) and immune checkpoint inhibition (ICI). However, disease characteristics and patient-specific risk factors of PTP after combined R(C)T + ICI are less understood. Given that RT-triggered PTP is strongly dependent on the volume and dose of RT [1], driven by inflammatory mechanisms, we hypothesize that combination therapy of R(C)T with ICI influences the dose-volume-effect correlation for PTP. This study focuses on the development of a method for evaluation of alterations of dosimetric parameters for PTP after R(C)T with and without ICI.

**Methods and materials:**

PTP volumes were delineated on the follow-up diagnostic Computed Tomography (CT) and deformably matched to the planning CT for patients with PTP after thoracic R(C)T + ICI or R(C)T. Dose data was converted to 2-Gy equivalent doses (EQD2) and dosimetrically analyzed. Dosimetric and volumetric parameters of the segmented PTP volumes were analyzed. The method was exemplarily tested on an internal patient cohort including 90 patients having received thoracic R(C)T + ICI (39) and R(C)T (51). Thirtytwo patients with PTP were identified for further analysis. Additional data on previous chemotherapy, RT, smoking status and pulmonary co-morbidity were conducted. A matched pair analysis with regard to planning target volumes (PTV) was conducted for curative intended (definitive) and palliative patient cohorts individually.

**Results:**

The presented method was able to quantify and compare the dosimetric parameters of PTP for the different therapies. For our study group, no significant differences between R(C)T + ICI and R(C)T only was observed. However, the dosimetric analysis revealed large volumetric fractions (55%) of the PTP volumes to be located outside of high dose (EQD2 < 40 Gy) regions for R(C)T + ICI. There was a non-significant trend towards increased area under the curve of the dose volume histogram (AUC) values for R(C)T + ICI compared to R(C)T only (3743.6 Gy∙% vs. 2848.8 Gy∙%; *p*-value = 0.171). In contrast to the data for the palliative intended treatment group, for definitive R(C)T + ICI, data tended towards increased volumes with higher doses.

**Conclusions:**

The proposed method was capable to quantify dosimetric differences in the dose-volume-effect relationship of PTP for patients with R(C)T + ICI and patients with R(C)T only. In this exploratory analysis, no significant dosimetric differences within PTP volumes for the different groups could be observed. However, our observations suggest, that for safe application of thoracic R(C)T + ICI, further careful investigation of dosimetric prescription and analysis concepts with larger and conformer study groups is recommendable.

## Background

Immune checkpoint inhibitors (ICIs) such as programmed cell death-ligand 1 (PD-L1) and programmed cell death-1 (PD-1) have altered the clinical treatment landscape for lung cancer due to unprecedented improved clinical outcome. Due to convincing results with improved survival after radio(chemo)therapy (R(C)T) [2], the PD-L1 inhibitor durvalumab is routinely applied for unresectable, stage III non-small cell lung cancer (NSCLC) after RCT as maintenance therapy. Further application, e.g. for metastatic disease is growing, leading to an increase in the use of ICI therapy with radiotherapy (RT) [3].

Post-therapy pneumonitis (PTP) as a relevant and potentially fatal side effect of both, RT and ICI, limits the applicable dose and challenges the therapeutic efficacy [4]. Usually, radiation induced pneumonitis occurs 4 to 12 weeks after RT and is restricted to the radiation field. The incidence largely varies between 13 and 36% depending on the dose regime and the method of follow-up applied in the presenting studies [5]. RT can enhance the immunogenic effect by up-regulation of PD-L1 and PD-1 resulting in an increased anti-tumoral response [6, 7], which may result in an increased therapeutic effect, but also in an altered normal tissue response. Whereas the majority of existing data does not show an increase of severe pulmonary toxicity for combined radioimmunotherapy, the incidence of pneumonitis over all grades seems to be increased [8–14].

However, no conclusion with regard to dose and fractionation schemes can be drawn from these results. This is of major importance for RT dose prescription and treatment planning. As PTP might originate from both, RT and ICI therapy, it is reasonable to reconsider existing dose-volume-effect correlations. Only a small number of studies focus on this topic [15, 16]. Data on PTP after stereotactic body radiation therapy (SBRT) and ICI therapy suggests consisting dose constraints to be safe [16].

In this work, we present a method to explore dosimetric parameters of PTP after normofractionated R(C)T + ICI therapy aiming to generate a hypothesis for further clinical investigations.

## Methods

### Dosimetric analysis

Patient CT-scans with a slice thickness of 0.9–3 mm showing pneumonitis at the time of first occurrence were analyzed. The treatment planning software Eclipse versions 15.6 and 16.0 (Varian Medical Systems, Palo Alto, Santa Clara, CA, USA) was used to accurately delineate the volume encompassing the radiological extensions of the pneumonitis. The derived contours were validated and approved by experienced specialists in radiology and nuclear medicine. Contours were transformed to the RT planning CT using deformable image registration applying a demon’s algorithm [17]. In case of overlap with the gross tumor volume (GTV), the GTV was subtracted from the pneumonitis contours to ensure solid tumor mass not to contribute to the assessment of pneumonitis. Three dimensional voxel-wise dose data was converted to 2 Gy equivalent doses (EQD2) based on the Linear Quadratic Model (LQM) [18] using a Matlab (MATLAB R2019b, The MathWorks Inc., Natick, MA, USA) [19] script. An α/β ratio of 3 for normal lung tissue was assumed [20]. For the pneumonitis volume, relevant dosimetric data was extracted such as the volume fraction receiving at least 20 Gy (V_20Gy_) and the volume receiving 20 Gy in cm^3^ as a measure for rather low dose volumes, mean dose as an intuitive measure for the received dose, as well as the volume fraction receiving at least 40 Gy (V_40Gy_) as a measure for the volumetric fraction receiving higher doses. Dose data was categorized into 3 dose levels: low dose (LD) comprising doses below 20 Gy, intermediate dose (ID) comprising doses ranging from 20 Gy to 40 Gy and high dose (HD) with a minimum dose of 40 Gy. DVHs were extracted and the area under the curve (AUC) of the DVHs was derived. For the total lung, the original mean lung dose (MLD) and the V_20Gy_ were extracted.

### Patient data

Our method was tested using patient data as depicted in Table 1. Ninety patients, who received thoracic R(C)T with (39) or without ICI (51) in a time interval of 110 days around R(C)T between 2010 and 2022 at our institute were collected. Data was conducted based on patient data files and imaging data. Patient follow-up after definitive treatment included clinical examination and chest CT scans 6 weeks after therapy and every 3 to 6 months for 3 years, every 6 months for 2 years followed by once yearly intervals. Follow-up schedules after palliative treatment were based on a patient individual basis.

**Table 1:** Patient characteristics., ^1^R(C)T abbreviates radio(chemo)therapy, ^2^ICI stands for immune checkpoint inhibition ^3^CTx stands for chemotherapy, ^4^SD abbreviates standard deviation. ^5^MWU stands for Mann-Whitney-U test

Patient characteristics						
	R(C)T^1^ + ICI^2^	R(C)T + ICI [%]	R(C)T	R(C)T [%]	*p*-value	Test
No. of patients	39	43	51	56		
No. females	14	35.9	11	21.6	0.159	Chi-square
No. males	25	64.1	40	78.4	0.159	Chi-square
Median Age [a] (min; max)	69 (47;83)		62 (49;85)		0.058	MWU^5^
Pulmonary Co-morbidity	15	38.5	14	27.5	0.268	Chi-square
Active or former smokers	25	64.1	34	66.7	0.483	Chi-square
No. of patients with lung metastases	2	5.1	1	2.0	0.407	Chi-square
No. of patients with primary lung tumors	36	92.3	50	98.0	0.191	Chi-square
CTx^3^	35	89.7	37	72.5	0.043	Chi-square
concomitant CTx	16	41.0	16	31.4	0.343	Chi-square
Prior thoracic RT	2	5.1	1	2.0	0.191	Chi-square
Definitive R(C)T	25	64.1	36	70.6	0.516	Chi-square
Median time between ICI & RT (min; max) [d]	14 (0;76)		-			
No. of pneumonitis	16	41.0	16	31.4	0.578	Chi-square
Pneumonitis Grade CTCAE 1	10	62.5	12	66.7		
Pneumonitis Grade CTCAE 2	5	31.3	2	11.1		
Pneumonitis Grade CTCAE 3	1	6.3	2	11.1		
Mean onset time after RT (SD^4^) [d]	100.0 (49.73)		74.9 (59.97)		0.102	MWU
Median onset time after RT (min; max) [d]	87 (14;190)		54 (0;198)		0.102	MWU

The time interval between R(C)T and ICI therapy varied between 0 and 76 days.

Additional chemotherapy was administered in 35 (89.7%) cases in the R(C)T + ICI group and in 37 cases (72.5%) in the R(C)T only group. Three patients had a history of thoracic RT, 1 in the R(C)T only group within a time interval of more than 3 years and 2 in the R(C)T + ICI group with minimum of 11 months prior to radioimmunotherapy.

In total, 59 patients (65%) were former or active smokers, 25 (64.1%) in the R(C)T + ICI group and 34 (66.7%) in the R(C)T group. Twenty-nine patients suffered from pulmonary comorbidities, 15 (38.5%) in the R(C)T + ICI and 14 (27.5%) in the R(C)T only group.

#### Pneumonitis definition

Pneumonitis was diagnosed based on clinical and/or radiological findings and was graded according to the Common Terminology Criteria for Adverse Events (CTCAE) v5 [21]. All grades were included. Clinical symptoms covered coughing, dyspnea and thoracic pain. Radiological findings encompassed a variety of findings such as cryptogenic organizing pneumonia (COP), with ground-glass and consolidative opacities. Nonspecific interstitial pneumonia (NSIP), another form of interstitial lung disease, presents with ground-glass and reticular opacities, indicating thickening of the interstitial lung tissue [12, 22–24].

### Statistical analysis

Exploratory statistical analysis was performed using IBM SPSS Statistics version 28.0.1.1 (14). Univariate analysis and analysis of significance was performed using chi-squared tests for categorial variables. For numeric data, we applied Mann-Whitney-U (MWU) tests. Statistical significance level was set at *p* < 0.05.

In a first step, statistical analysis was performed for the entire data set. In a second step, the data set was divided into two groups of patients to reduce the impact of biologically different dose schemes. One group contained patients, who received definitive R(C)T ± ICI, and the other group summarized patients who received palliative R(C)T ± ICI. Cases in these subgroups were matched pairwise according to their planning target volumes (PTVs) in order to reduce the interfering influence of non-matching irradiated volumes on the radiation dose-volume correlation.

## Results

### Dosimetric data analysis across all cases

We introduced a method applicable for evaluation of potential differences in dosimetric parameters of PTP when additional ICI is administered to R(C)T. Application of this method to a small test cohort did not reveal significant differences in the tested parameters between the two study groups. Our results imply large volumetric fractions of PTP (55%) to be located outside of the high dose RT field for R(C)T + ICI. In contrast to palliative intended treatment, a trend towards larger PTP volumes with higher doses could be observed for combined definitive treatment.

We exploratorily investigated 90 patients having received R(C)T + ICI (39) or R(C)T alone (51) as summarized in Table 1. Eighty-six patients with primary lung cancer and 3 patients with lung metastases and one with pleural carcinomatosis were included. RT fractionation schemes varied with total doses from 30 to 66 Gy and single doses between 1.8 Gy and 3.0 Gy. Sixty-one patients received definitive (meaning curatively intended) R(C)T +/- ICI and 29 patients were treated in palliative intention as listed in Table 1. From the 39 patients, who received ICI therapy, all were treated with PD-L1 or PD-1 inhibitors. Out of the group receiving ICI therapy, the majority of 23 patients (59%) received Durvalumab.

**Fig. 1:**
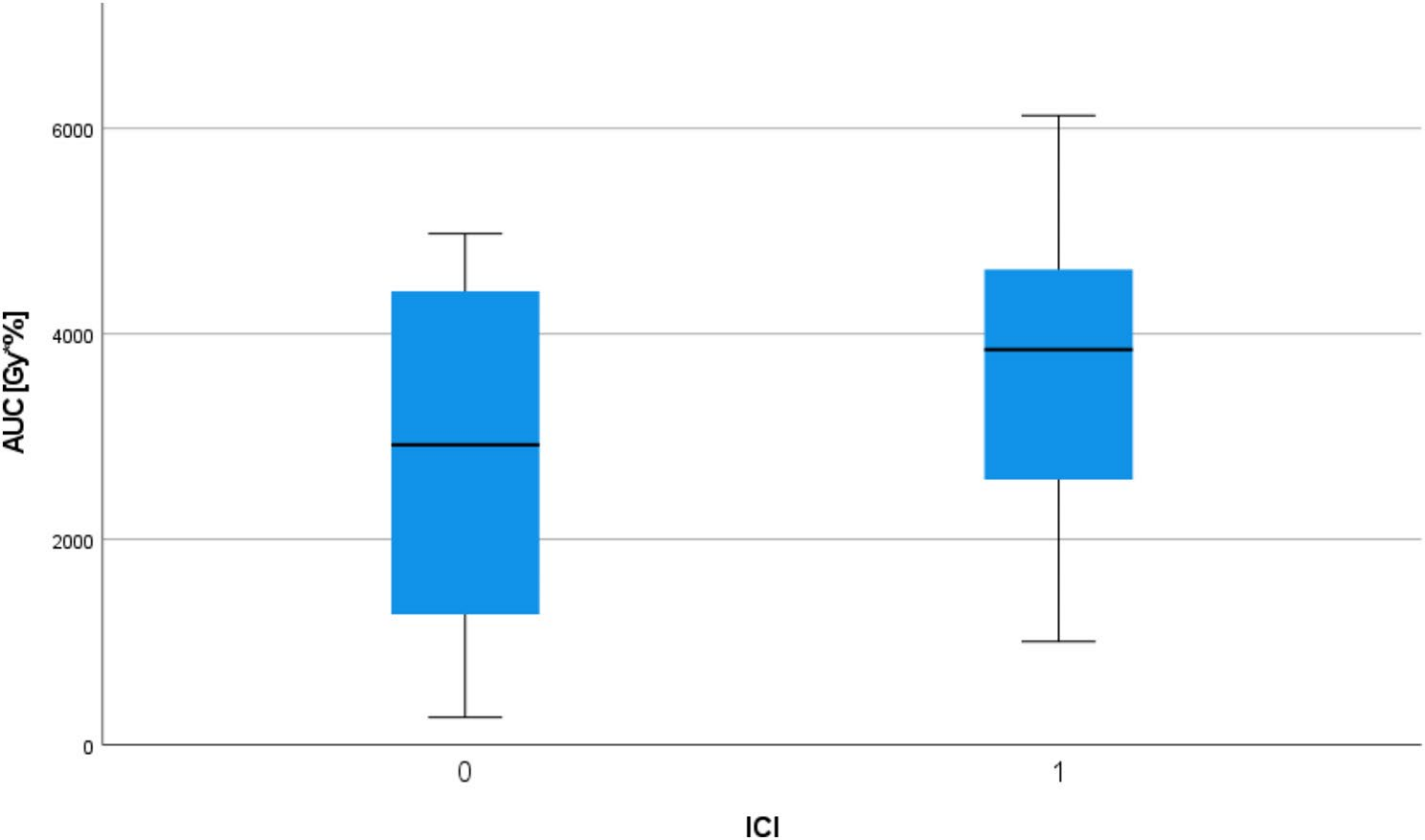
Boxplot of pneumonitis AUC values for all patients with and without additional immune checkpoint inhibition (ICI)

In total, 32 patients were diagnosed with any grade pneumonitis and are depicted in Tables 2, 16 (41%) in the R(C)T + ICI group and 16 (31.4%) in the R(C)T group. Mean EQD2 PTP doses were numerically increased for R(C)T + ICI (35.9 vs. 28.8, *p* = 0.239) and a pronounced pneumonitis volume fraction of 45% could be observed in the HD region (45% vs. 33.8%; *p* = 0.341) and a small pneumonitis volume fraction of 26% in the LD region (26% vs. 39.7%, *p* = 0,451), however, without statistical significance. The same applies for the DVH analysis, which did not reveal statistically significant difference of the AUC values for R(C)T + ICI (3743.6 Gy∙% vs. 2848.8 Gy∙%, *p* = 0.171). However, as depicted in Fig. 1, numerically, AUC values differ, even though MLD and V20_total lung_ were comparable between both groups (MLD 11.5 Gy vs. 12 Gy; *p* = 0.926, RCT + ICI vs. RCT; V20_total lung_ 17.4% vs. 18.6; *p* = 0.956, RCT + ICI vs. RCT).

The mean onset time of PTP after treatment was increased after R(C)T + ICI (100.0 days vs. 74.9 days; *p* = 0.102).

**Table 2:** Dosimetric parameters for all radio(chemo)therapy ^1^R(C)T ± immune checkpoint inhibition (^2^ICI) patient cohorts. ^3^SD stands for standard deviation; ^4^Min and Max abbreviates minimum and maximum values; ^5^AUC stands for area under the curve of the dose volume histogram (DVH) for the pneumonitis volume; ^6^PTV abbreviates planning target volume

		*R*(C)T^1^ + ICI^2^	*R*(C)T	*p*-value	Test
**Number of patients**		16	16		
V_pneumonitis_ [cm^3^]	Mean	216.4	249.9	0.838	MWU
	SD^3^	305.6	300.0		
	Median	94.5	135.0		
	Min^4^	10.0	8.9		
	Max^4^	1147.0	1126.7		
V20_pneumonitis_ EQD2 [cm^3^]	Mean	90.7	138.6	0.752	MWU
	SD	95.7	186.2		
	Median	55.0	70.7		
	Min	9.8	2.0		
	Max	350.2	708.6		
Mean EQD2_pneumonitis_ [Gy]	Mean	35.9	28.8	0.239	MWU
	SD	12.4	14.4		
	Median	36.9	28.6		
	Min	10.1	6.6		
	Max	58.2	49.8		
High dose volume fraction [%]	Mean	45.0	33.8	0.341	MWU
	SD	30.4	25.6		
	Median	43.1	32.7		
	Min	4.9	0.1		
	Max	99.2	76.2		
Intermediate dose volume fraction [%]	Mean	35.6	36.1	0.699	MWU
	SD	28.4	20.3		
	Median	28.3	30.7		
	Min	0.4	4.6		
	Max	98.2	86.4		
Low dose volume fraction [%]	Mean	26.0	39.7	0.415	MWU
	SD	26.2	31.0		
	Median	14.7	32.6		
	Min	0.4	0.1		
	Max	85.5	95.4		
AUC^5^ [Gy*%]	Mean	3743.6	2848.8	0.171	MWU
	SD	1395.6	1529.9		
	Median	3848.1	3002.0		
	Min	1006.6	268.4		
	Max	6126.3	4976.9		
MLD_total lung_ EQD2 [Gy]	Mean	11.5	12.0	0.926	MWU
	SD	3.9	3.5		
	Median	12.8	12.6		
	Min	3.8	5.4		
	Max	18.3	17.9		
V20_total lung_ EQD2 [%]	Mean	17.4	18.6	0.956	MWU
	SD	6.5	7.4		
	Median	17.4	18.7		
	Min	5.5	8.6		
	Max	28.0	35.4		
PTV^6^ [cm^3^]	Mean	495.0	443.2	0.669	MWU
	SD	273.4	269.2		
	Median	419.5	407.5		
	Min	92.7	117.6		
	Max	1125.6	1068.7		

### Matched pair analysis

Groups were separated into patients, who were treated in definitive or palliative intention. Definite total doses ranged from 54 Gy to 66 Gy delivered in single dose fractions from 1.8 to 2 Gy. Palliative patients received variable dose schemes including single doses between 1.8 Gy up to 3 Gy and total doses up to 50.4 Gy. All results are summarized in Table 3.

**Table 3:** Dosimetric parameters for statistical analysis matched on the planning target volume (^1^PTV) patient cohorts for definitive and palliative radio(chemo)therapy ^2^R(C)T ± immune checkpoint inhibition (^3^ICI) patient cohorts. ^4^SD stands for standard deviation; ^5^Min and Max abbreviates minimum and maximum values; ^6^AUC stands for area under the curve of the dose volume histogram (DVH) of the pneumonitis volume

Matched Pair Analysis					
	Definitive R(C)T^2^			Palliative R(C)T	
Matched Parameter	PTV^1^[cm^3^]				
		R(C)T + ICI^3^	R(C)T	R(C)T + ICI	R(C)T
**Number of Patients**		6	6	4	4
V_pneumonitis_ [cm^3^]	Mean	218.2	141.9	95.5	229.6
	SD^4^	252.2	189.6	117.6	118.4
	Median	160.0	53.1	55.4	239.3
	Min^5^	27.0	18.7	10.0	86.9
	Max^5^	703.2	505.6	261.2	352.9
V20_pneumonitis_ EQD2 [cm^3^]	Mean	123.4	85.8	38.7	88.6
	SD	123.2	97.5	22.6	26.5
	Median	79.3	41.4	40.2	82.0
	Min	24.4	2.0	9.8	64.7
	Max	350.2	222.6	64.8	125.8
Mean EQD2_pneumonitis_ [Gy]	Mean	39.9	28.6	29.6	23.0
	SD	11.2	16.2	15.6	9.9
	Median	39.1	30.5	30.9	23.5
	Min	23.9	6.6	10.1	11.9
	Max	58.2	47.0	46.4	33.0
High dose [%]	Mean	55.1	66.9	24.3	27.4
	SD	25.6	37.1	38.0	20.2
	Median	49.2	79.1	5.4	27.0
	Min	25.6	4.6	4.9	7.0
	Max	99.2	99.9	81.4	48.5
Intermediate dose [%]	Mean	35.6	39.4	49.6	38.6
	SD	23.9	27.0	40.3	18.6
	Median	35.2	36.7	44.4	38.2
	Min	0.4	4.6	11.6	20.4
	Max	72.8	86.4	98.2	57.8
Low dose [%]	Mean	16.5	33.1	32.4	51.4
	SD	18.1	37.1	37.9	26.9
	Median	8.9	20.9	21.1	50.9
	Min	0.4	0.1	1.8	25.5
	Max	50.2	95.4	85.5	78.3
AUC^6^ [Gy*%]	Mean	3988.4	2989.6	2958.0	1994.5
	SD	1124.7	1495.4	1558.2	1508.1
	Median	3913.0	3046.9	3091.5	2202.6
	Min	2386.6	660.1	1006.6	268.4
	Max	5824.3	4687.8	4642.3	3304.3
MLD_total lung_ EQD2 [Gy]	Mean	12.0	12.8	6.7	9.2
	SD	1.8	2.4	2.8	3.5
	Median	12.8	12.9	6.2	9.5
	Min	9.5	10.2	3.8	5.4
	Max	13.6	16.9	10.5	12.6
V20_total lung_ EQD2 [%]	Mean	20.4	19.4	9.5	13.5
	SD	4.4	4.6	5.2	4.9
	Median	21.2	19.7	7.8	12.8
	Min	15.0	12.6	5.5	8.6
	Max	27.1	26.4	16.9	19.7
PTV [cm^3^]	Mean	539.3	531.9	333.0	320.2
	SD	300.4	284.4	214.1	183.4
	Median	446.4	463.3	324.9	304.4
	Min	293.5	250.6	92.7	117.6
	Max	1125.6	1068.7	589.4	554.3

#### Definitive treatment

**Fig. 2:**
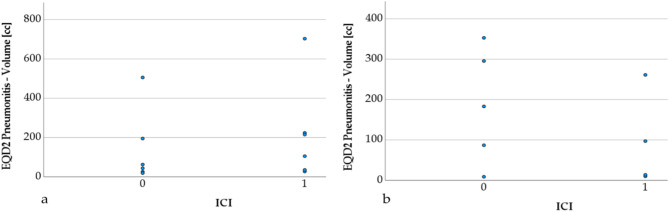
Scatter plots for pneumonitis volumes for the definitive (**a**) and palliative (**b**) radio(chemo)therapy R(C)T patient cohort with and without immune checkpoint inhibition (ICI). Opposing trends between palliative and definitive treatments can be observed

**Fig. 3:**
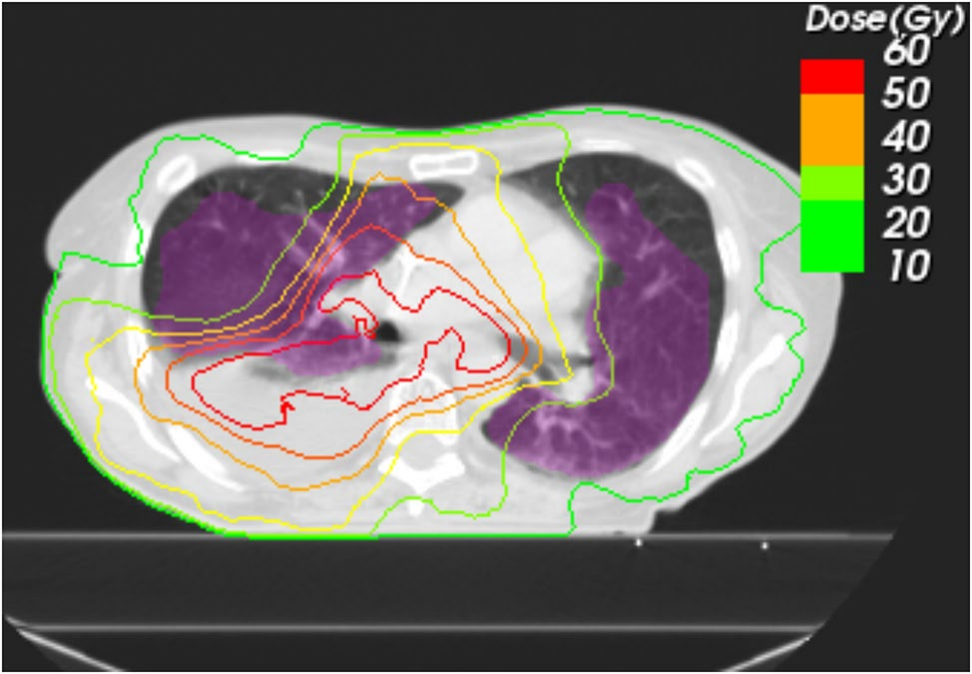
Axial Computed Tomography (CT) scan with EQD2 isodose lines and the pneumonitis contours matched in color-washed magenta. The majority of the pneumonitis volume is located outside the high dose region

**Fig. 4:**
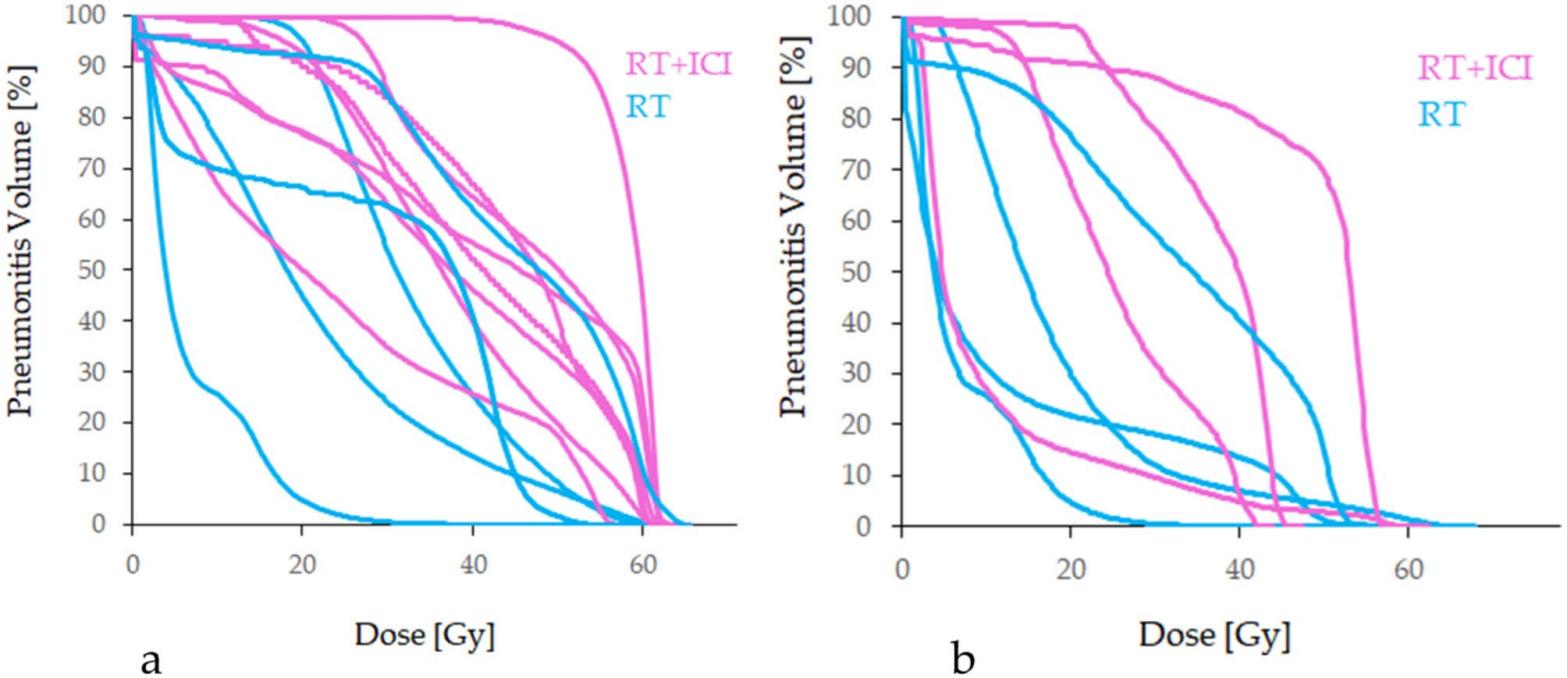
DVHs for each pneumonitis volume for definitive (**a**) and palliative (**b**) R(C)T with ICI (magenta) and without ICI (blue). For definitive treatment, a shift to the right can be noticed for R(C)T + ICI

Six patients with pneumonitis, who received definitive R(C)T + ICI were matched according to their PTVs to 6 patients in the R(C)T group (see Table 3). Due to very small sample sizes, no significance tests were performed. Similar numerical trends as for the overall patient cohort were observed. PTP volumes were large (218.2 cm^3^ vs. 141.9 cm^3^) with large fractions in the HD regions (55.1% vs. 66.9%) and increased AUC values (3988.4 Gy∙% vs. 2989.6 Gy∙%). PTP volumes for definitive and palliative R(C)T with and without ICI are depicted in Fig. 2. An exemplary CT scan from a patient’s lung after definitive R(C)T + ICI in Fig. 3 shows the extension of the pneumonitis beyond the HD region of the radiation field. Figure 4 shows the DVHs for definitive and palliative R(C)T with and without ICI. For definitive treatment, a shift towards higher doses with increased volumes resulting in higher AUC values can be observed.

#### Palliative treatment

In this group, 4 patients with pneumonitis in the R(C)T + ICI group were matched according to their PTVs to 4 patients with pneumonitis in the R(C)T group. Numerical trends within this group do not match the findings for the definitive and overall treatment group. PTP volumes (95.5 cm^3^ vs. 229.6 cm^3^) and V20 of the PTP (38.7 cm^3^ vs. 88.6 cm^3^) were smaller.

## Discussion

We introduced a method to evaluate and compare dosimetric differences of PTP volumes between thoracic R(C)T with and without additional immunotherapy. The results from our statistical analysis suggest there are no differences in the dosimetric parameters when additional ICI therapy is added to R(C)T. However, exploratory application of the proposed method suggests that there might be quantitative numerical difference of PTP volumes for combined radioimmunotherapy compared to R(C)T only without statistical significance, however.

The proposed PTP evaluation method is based on well-established DVH data analysis and thus can be easily reproduced with conventional treatment planning systems. We provided and compared dosimetric analysis of PTP and total lung parameters to reveal potential influences of the lung dose distribution to PTP extension. We defined reasonable dose levels assisting first glance evaluation of the PTP extension with respect to the radiation field. The method applies diagnostic thoracic CT scans, that are acquired in the course of follow up visits anyway, ensuring no additional radiation is administered to the patient and no additional examination is required. One limitation of the applied method is the dependence of user defined segmentation of the PTP contours. In this study, we tried to minimize this impact by independent radiological expert approval of the delineated contours. For future improvement of the method, automatic segmentation by atlas-based algorithms or by application of artificial intelligence could be implemented, also helping to improve the performance of the process.

Our results suggest that additional ICI therapy does not influence the dose-volume-effect for pneumonitis after RT. However, the data can show numerical trends towards large PTP volumes and increased AUC values after combined radioimmunotherapy without statistical significance. Our data sample was too small and inhomogeneous to result in significant results and should be validated with a larger data set. Numerically larger AUC values without statistical significance (*p*-value of 0.171) after combined R(C)T + ICI (3743.6 Gy∙% vs. 2848.8 Gy∙%) for the overall cohort were found. Even after matched pair analysis and differentiation between definitive and palliative treatment, no clear numerical trends for the groups were found. Whereas mean PTP volumes seemed numerically increased for R(C)T + ICI in definitive treatment intention (218.2 cm^3^ vs. 141.9 cm^3^) with increased mean EQD2 and V20_pneumonitis_ to the PTP, after palliative R(C)T + ICI, opposingly, PTP volumes were smaller (95.5 cm^3^ vs. 229.6 cm^3^) and mean PTP doses and V20_pneumonitis_ were smaller. These results could indicate differences comparing palliative and definitive treatment, however, these differences between the groups might be due to small groups and statistical fluctuation. Another explanation for smaller PTP volumes in the palliative group might be that the majority of definitive treatments were due to primary lung cancers, where the additional ICI therapy lead to activation of immunogenic systemic response causing an extension of the pneumonitis volumes, whereas in the palliatively treated group, mediastinal treatment was more common and total prescription doses were smaller resulting in less actual dose to the lung tissue. One case in the palliative treatment group, who received ICI therapy showed a large overlap between the initial GTV and the pneumonitis volume resulting in a methodologically reduced pneumonitis volume influencing the analysis towards smaller pneumonitis volumes. Thus, results for palliative treatment have to be evaluated cautiously.

Surely, our results suffer from multiple limitations. With regard to the method itself, deformable image registration is applied which introduces inaccuracy depending on the algorithm and potential differences in image quality. In this work, the same algorithm was used for all data and CT scans for RT dose calculation were performed at the same CT scanner reducing part of the introduced error. Surely, quality of the follow up CTs might differ and thus might influence registration performance. Besides the retrospective design of the study, firstly, the very small size of the study cohort of 32 patients with pneumonitis limits the statistical meaningfulness. Our results are majorly statistically negative, which might be due to this small sample size. Secondly, the group characteristics are rather inhomogeneous with respect to dose regimes, target volume sizes and treatment techniques. Trying to smooth these inhomogeneities with a matched pair analysis, resulted in a too small sample size for statistical analysis, unfortunately.

Data on dosimetric parameters of PTP volumes for combined radioimmunotherapy using ICIs is sparse. Watanabe et al. investigated dose relationships for pneumonitis after definitive RCT followed by durvalumab maintenance therapy and found lower pneumonitis volume fractions receiving minimum doses of 5 Gy (V_5Gy_) to 50 Gy (V_50Gy_) for grade 2 pneumonitis compared to grade 1 pneumonitis. Based on their findings, the authors suggest the 15-Gy isodose line as a definition of the radiation field responsible for pneumonitis [15]. Voong et al. studied the relationship between thoracic RT and development of PTP in NSCLC patients, who received ICI therapy. They found overall increased PTP rates of 19%. Patients, who were treated in curative intent with median total doses up to 60.5 Gy were more likely to develop pneumonitis compared to palliatively treated patients with doses up to 30 Gy (17/19, 89% vs. 2/19, 11%; *p* = 0.051). The spreading of radiological pneumonitis appearances were mostly found outside intermediate (20 Gy < D < 40 Gy) and high dose (D > 45 Gy) RT regions [25]. Compared to our findings, we rather found PTP within the HD and ID level. Including all data and for the palliative R(C)T + ICI group, we observed intermediate doses to contribute the most to the radiological findings. Whereas Voong et al. included patients with any previous RT and differentiated between more or less than 1-year interval between RT and ICI treatment, in our study, we focused on combined treatment with a time interval of up to 110 days. One reason for this choice of time interval was to consider rather acute and subacute immunologic effects. The other reason was to avoid interfering effects that inevitably arise with time due to potential additional sequential treatments.

Part of the effect leading to large pneumonitis volumes might be due to immune-related effects linked to an altered tumor microenvironment caused by RT. Across all groups, we observed a mild trend towards a delayed onset of pneumonitis after radioimmunotherapy (100 days vs. 75 days, *p* = 0.102). The incidence after ICI therapy has been studied and median onset time to ICI caused pneumonitis was found to be 82 days after initiation of ICI therapy [26], which is in the range observed here. In two case studies, also a delayed PTP onset of 5 months and 167 days after radioimmunotherapy were observed [27, 28]. However, the difference in timing between the investigated groups in this study, suggest that PTP occurrence after combined radioimmunotherapy is influenced by altered effects compared to radiation induced PTP. While therapy using ICIs has revolutionized cancer treatment with unprecedented survival, immune enhancement through ICI therapy administered directly after RT might increase the risk for immune-related side effects such as pneumonitis and can be the reason for delayed onset of PTP.

This study was focused on the establishment of a valid and reproducible method to analyze dosimetric differences for PTP after R(C)T with and without ICI and our results demonstrated its feasibility. While the dosimetric findings contribute to the rare results on dose-volume relationship for PTP after combined radioimmunotherapy, the application of the proposed method to our dataset is limited by the retrospective design, the small and inhomogeneous patient cohort combined with the rather rare event of PTP, that failed to approach the pre-defined significance level, restricting the conclusions.

## Conclusions

We introduced a valid and easily reproducible method for analysis of dosimetric parameters of PTP after thoracic radio(immuno)therapy. This method can help to explore or rule out potential dosimetric changes after thoracic R(C)T, that might be triggered by additional ICI therapy. Testing our method on a small patient cohort, the results suggest no impact of additional ICI therapy on the dose-volume-effect for the development of PTP. To validate these results and to rule out potential associations, that might have been obscured by the limited sample size, the proposed method should be applied to a larger and more homogeneous dataset.

## Data Availability

No datasets were generated or analysed during the current study.
